# Interpretable machine learning model for early prediction of delirium in elderly patients following intensive care unit admission: a derivation and validation study

**DOI:** 10.3389/fmed.2024.1399848

**Published:** 2024-05-17

**Authors:** Dayu Tang, Chengyong Ma, Yu Xu

**Affiliations:** Department of Critical Care Medicine, West China Hospital, Sichuan University, Chengdu, China

**Keywords:** elderly, delirium, ICU, prediction model, explainable machine learning

## Abstract

**Background and objective:**

Delirium is the most common neuropsychological complication among older adults admitted to the intensive care unit (ICU) and is often associated with a poor prognosis. This study aimed to construct and validate an interpretable machine learning (ML) for early delirium prediction in older ICU patients.

**Methods:**

This was a retrospective observational cohort study and patient data were extracted from the Medical Information Mart for Intensive Care-IV database. Feature variables associated with delirium, including predisposing factors, disease-related factors, and iatrogenic and environmental factors, were selected using least absolute shrinkage and selection operator regression, and prediction models were built using logistic regression, decision trees, support vector machines, extreme gradient boosting (XGBoost), k-nearest neighbors and naive Bayes methods. Multiple metrics were used for evaluation of performance of the models, including the area under the receiver operating characteristic curve (AUC), accuracy, sensitivity, specificity, recall, F1 score, calibration plot, and decision curve analysis. SHapley Additive exPlanations (SHAP) were used to improve the interpretability of the final model.

**Results:**

Nine thousand seven hundred forty-eight adults aged 65 years or older were included for analysis. Twenty-six features were selected to construct ML prediction models. Among the models compared, the XGBoost model demonstrated the best performance including the highest AUC (0.836), accuracy (0.765), sensitivity (0.713), recall (0.713), and F1 score (0.725) in the training set. It also exhibited excellent discrimination with AUC of 0.810, good calibration, and had the highest net benefit in the validation cohort. The SHAP summary analysis showed that Glasgow Coma Scale, mechanical ventilation, and sedation were the top three risk features for outcome prediction. The SHAP dependency plot and SHAP force analysis interpreted the model at both the factor level and individual level, respectively.

**Conclusion:**

ML is a reliable tool for predicting the risk of critical delirium in elderly patients. By combining XGBoost and SHAP, it can provide clear explanations for personalized risk prediction and more intuitive understanding of the effect of key features in the model. The establishment of such a model would facilitate the early risk assessment and prompt intervention for delirium.

## Introduction

Delirium, also known as acute encephalopathy, is a neuropsychiatric syndrome characterized by acute changes or fluctuations of cognitive function, inattention, disorganized thinking, and altered level of consciousness (1, 2). Delirium is highly prevalent among hospitalized older adults and represents the most common neuropsychological complication in older patients within the intensive care unit (ICU) (3). Reported incidence rates of delirium among hospitalized older adults ranges from 14 to 56%, depending on patient population and screening instrument (4–6). In the ICU, the prevalence of delirium has been shown to reach as high as 60–80% (3, 7). Delirium in older patients often arises due to a complex interplay of factors exacerbating challenges posed by the ICU environment, including prolonged mechanical ventilation (MV) and hospital stay, increased costs, long-term cognitive impairment, and increased risk of death (8, 9).

It is now known that antipsychotics and other psychoactive medications do not reliably improve brain function in critically ill patients with delirium (10). According to the 2018 Pain, Agitation/Sedation, Delirium, Immobility, and Sleep Disorders in Adult Patients in the ICU Guideline, clinicians need to pay increased attention to the screening of high-risk delirium patients and actively implementing approaches to prevent delirium (11). Therefore, a reliable delirium predictive model will help clinicians identify delirium high-risk patients and guide timely interventions. In fact, several predictive models have been developed for delirium in the ICU, including the PRE-DELIRIC model, the E-PRE-DELIRIC model, and the DYNAMIC-ICU model (12–15). However, all of these models were based on results from a wide range of age groups and did not take into consideration the characteristics of older patients. There are other alternative models available for predicting delirium in older adults, but these models have been mainly validated in postoperative individuals, and their applicability to ICU patients is still uncertain (16–19). Therefore, there is still a lack of delirium risk prediction models applicable to older patients admitted to the ICU.

Compared to traditional regression analysis, machine learning (ML) methods offer numerous potential advantages for studies of older adults (20). With the abundance of data available from geriatric cohort studies and electronic health records, ML methods can enhance the accuracy and efficiency of prediction models in aging applications while leveraging the increasing amounts of health system data (21). However, due to the “black box” of ML algorithms, this makes it difficult to understand the predicted outcomes and limits the applications of these models (18). Notably, the SHapley Additive exPlanation (SHAP) methods have gained increasing prominence in addressing this issue (19). SHAP has significant advantages in elucidating how the ML model calculates the features required for prediction and visualizing the prediction models. It has been successfully applied to improve clinical understanding of a variety of diseases, including the risk of hypoxemia during surgery, the prognosis of acute kidney injury, and the risk factors for sepsis and septic death (22–24). However, there is currently no interpretable ML method to predict the risk of delirium in critically ill older patients.

The objective of this study was to develop and validate a predictive model for delirium in ICU patients aged 65 years and older using six ML algorithms. In addition, the SHAP method was used to provide a comprehensive explanation and enhancing clinical understanding for the best performing model. The findings from this study would facilitate early identification of high-risk older individuals prone to delirium in ICU settings, thereby enabling clinicians to implement timely interventions.

## Materials and methods

### Data source

The study was conducted using the extensive electronic health record database of the Medical Information Mart for Intensive Care (MIMIC)-IV version 2.2 (v2.2). Specifically, the MIMIC database contains comprehensive and high-quality data on both deidentified and characterized adult patients (≥18 years old) who were admitted to the ICU at Beth Israel Deaconess Medical Center between 2008 and 2019 (25). MIMIC-IV v2.2 is the latest version of the MIMIC database, incorporating contemporary data (26). The institutional review board at MIT (Cambridge, MA) and Beth Israel Deaconess Medical Center (Boston, MA) approved the use of this database, granting a waiver of informed consent for this study while ensuring compliance with ethical standards outlined in the Declaration of Helsinki. One of our authors has been granted access to the database (CM, Certification Number: 34907227). Our study adhered to the Transparent Reporting of a Multivariable Prediction Model for Individual Prognosis or Diagnosis (TRIPOD) statement (27).

### Study population and outcome

Older patients were included if they met the following criteria: (1) admitted to the ICU; (2) underwent delirium assessment; (3) aged ≥65 years older. The assessment of delirium in the MIMIC-IV v2.2 database was conducted using the Confusion Assessment Method for the ICU (CAM-ICU) score. The CAM-ICU score is the most effective tool for diagnosing and assessing delirium in adult ICU patients according to the 2013 Society of Critical Care Medicine guidelines for pain, agitation, and delirium, which consists of four features: (1) an acute onset of mental status changes or a fluctuating course; (2) inattention; (3) disorganized thinking; and (4) an altered level of consciousness (28). Patients were diagnosed with delirium (i.e., CAM-ICU positive) if they presented with features 1 and 2, in addition to either feature 3 or 4. We excluded patients who had been hospitalized for less than 48 h and those already diagnosed with dementia, as the latter can be easily misdiagnosed as cognitive impairment. In cases where patients had multiple admissions to the ICU, only their first admission was analyzed.

This is a retrospective observational study in which all enrolled patients have undergone delirium assessment. They were further divided into two groups: delirious patients (case group) and non-delirious patients (control group), and a comparison of baseline characteristics between the two groups was conducted (see Table 1). The primary outcome of this study was the occurrence of delirium during ICU stay. All enrolled patients were followed from inclusion until ICU discharge, hospital discharge, or in-hospital death.

**Table 1:** Baseline characteristics of patients with and without delirium.

Variables	Total (*N* = 9,748)	Non-delirium (*N* = 5,505)	Delirium (*N* = 4,243)	*p*-value
Age (years)	76 (70, 83)	76 (70, 82)	76 (71, 83)	0.004
Male (%)	5,277 (54.1)	2,970 (54.0)	2,307 (54.4)	0.7
Ethnicity (%)				<0.001
Asian	1,688 (17.3)	841 (15.3)	847 (20.0)	
Black	765 (7.8)	390 (7.1)	375 (8.8)	
Hispanic	210 (2.2)	123 (2.2)	87 (2.1)	
White	6,665 (68.4)	3,900 (70.8)	2,765 (65.2)	
Others	420 (4.3)	251 (4.6)	169 (4.0)	
Marital Status (%)				<0.001
Single	2,476 (25.4)	1,278 (23.2)	1,198 (28.2)	
Married	4,771 (48.9)	2,801 (50.9)	1,970 (46.4)	
Divorced	658 (6.8)	378 (6.9)	280 (6.6)	
Others	1,843 (18.9)	1,048 (19.0)	795 (18.7)	
Admission type (%)				<0.001
Selective	1,538 (15.8)	1,025 (18.6)	513 (12.1)	
Urgent	7,850 (80.5)	4,244 (77.1)	3,606 (85.0)	
Emergent	360 (3.7)	236 (4.3)	124 (2.9)	
ICU type (%)				<0.001
CVICU	2,207 (22.6)	1,498 (27.2)	709 (16.7)	
CCU	1,405 (14.4)	941 (17.1)	464 (10.9)	
MICU	1,457 (14.9)	660 (12.0)	797 (18.8)	
M/SICU	1,317 (13.5)	735 (13.4)	582 (13.7)	
NICU	1,050 (10.8)	550 (10.0)	500 (11.8)	
SICU	1,286 (13.2)	629 (11.4)	657 (15.5)	
TSICU	1,026 (10.5)	492 (8.9)	534 (12.6)	
*Comorbidity*				
COPD (%)	1,133 (11.6)	541 (9.8)	592 (14.0)	<0.001
Hypertension (%)	4,638 (47.6)	2,698 (49.0)	1,940 (45.7)	0.001
Diabetes (%)	3,238 (33.2)	1,736 (31.5)	1,502 (35.4)	<0.001
Heart failure (%)	3,706 (38.0)	2,057 (37.4)	1,649 (38.9)	0.13
Atrial fibrillation (%)	4,397 (45.1)	2,396 (43.5)	2,001 (47.2)	<0.001
AMI (%)	1,448 (14.9)	776 (14.1)	672 (15.8)	0.017
CKD (%)	2,419 (24.8)	1,268 (23.0)	1,151 (27.1)	<0.001
Stroke (%)	2,112 (21.7)	990 (18.0)	1,122 (26.4)	<0.001
Tumor (%)	1,483 (15.2)	854 (15.5)	629 (14.8)	0.3
*Scoring system*				
GCS	15.0 (14.0, 15.0)	15.0 (14.0, 15.0)	14.0 (13.0, 15.0)	<0.001
APSIII	43 (33, 56)	39 (31, 51)	49 (37, 63)	<0.001
SAPS II	39 (32, 48)	37 (31, 44)	43 (36, 52)	<0.001
SOFA	5.0 (3.0, 7.0)	4.0 (2.0, 6.0)	6.0 (4.0, 9.0)	<0.001
*Vital signs*				
Heart rate (min^−1^)	81 (72, 92)	80 (72, 91)	83 (74, 95)	<0.001
Systolic BP (mmHg)	116 (106, 128)	115 (106, 128)	116 (106, 128)	0.2
Diastolic BP (mmHg)	59 (53, 67)	59 (53, 67)	59 (54, 67)	0.3
Mean BP (mmHg)	75 (70, 83)	75 (70, 83)	76 (70, 83)	0.11
Respiratory (min^−1^)	18.8 (16.8, 21.3)	18.6 (16.6, 21.0)	19.1 (17.0, 21.7)	<0.001
Temperature (°C)	36.8 (36.6, 37.1)	36.8 (36.6, 37.0)	36.9 (36.6, 37.2)	<0.001
*Lab. indicators*				
WBC (10^9^/L)	11.3 (8.4, 15.0)	10.9 (8.1, 14.5)	11.8 (8.9, 15.5)	<0.001
Hemoglobin (10^12^/L)	10.45 (9.15, 12.00)	10.45 (9.20, 12.00)	10.45 (9.05, 12.00)	0.2
Hematocrit (%)	32.0 (28.1, 36.7)	32.0 (28.2, 36.5)	32.2 (28.0, 36.8)	0.4
Platelet (10^9^/L)	180 (135, 239)	180 (136, 238)	180 (135, 241)	0.6
Bicarbonate (mmol/L)	23.0 (20.5, 25.0)	23.0 (21.0, 25.0)	22.5 (20.0, 24.5)	<0.001
Sodium (mmol/L)	138.5 (136.0, 141.0)	138.5 (136.0, 140.5)	139.0 (136.0, 141.5)	<0.001
Potassium (mmol/L)	4.20 (3.90, 4.60)	4.20 (3.90, 4.60)	4.20 (3.90, 4.65)	0.12
Chloride (mmol/L)	104.0 (100.0, 107.5)	104.0 (100.5, 107.0)	104.0 (100.0, 107.5)	0.5
Calcium (mmol/L)	8.40 (7.95, 8.85)	8.40 (7.98, 8.85)	8.35 (7.90, 8.80)	<0.001
Glucose (mg/dL)	132 (111, 163)	128 (109, 155)	138 (115, 173)	<0.001
BUN (mg/dL)	22 (16, 34)	21 (15, 31)	23 (17, 38)	<0.001
Creatinine (mg/dL)	1.05 (0.80, 1.55)	1.00 (0.75, 1.40)	1.10 (0.80, 1.75)	<0.001
Anion gap (mmol/L)	14.5 (12.5, 17.0)	14.0 (12.0, 16.0)	15.0 (13.0, 17.5)	<0.001
INR	1.25 (1.10, 1.50)	1.25 (1.10, 1.45)	1.30 (1.10, 1.55)	0.003
Prothrombin time (s)	13.8 (12.2, 16.3)	13.8 (12.2, 16.0)	13.9 (12.2, 16.8)	0.023
PTT (s)	32 (28, 42)	32 (28, 42)	32 (28, 41)	0.021
*ICU interventions*				
MV (%)	4,517 (46.3)	1,862 (33.8)	2,655 (62.6)	<0.001
RRT (%)	420 (4.3)	164 (3.0)	256 (6.0)	<0.001
Vasopressor use (%)	4,522 (46.4)	2,282 (41.5)	2,240 (52.8)	<0.001
Sedation (%)	5,239 (53.7)	2,345 (42.6)	2,894 (68.2)	<0.001
AKI (%)	7,007 (71.9)	3,699 (67.2)	3,308 (78.0)	<0.001
ICU-stay (days)	3.7 (2.6, 6.1)	3.1 (2.3, 4.2)	5.3 (3.3, 9.5)	<0.001
Hospital-stay (days)	9 (6, 15)	8 (5, 12)	12 (8, 20)	<0.001
ICU-mortality (%)	865 (8.9)	315 (5.7)	550 (13.0)	<0.001
Hospital-mortality (%)	1,486 (15.2)	551 (10.0)	935 (22.0)	<0.001

Data are presented as a number with the percentage in parentheses, or as the median with the interquartile range in parentheses. The “tumor” refers to a malignant cancer. Sedation includes midazolam, propofol, dexmedetomidine, and diazepam. ICU, intensive care unit; CCU, coronary care unit; CVICU, cardiovascular ICU; MICU, medical ICU; SICU, surgical ICU; NICU, neuro ICU; TSICU, trauma-neuro surgical ICU; COPD, chronic obstructive pulmonary disease; AMI, acute myocardial infarction; CKD, chronic kidney disease; GCS, Glasgow Coma Score; APSIII, the Acute Physiology Score III; SAPS II, the Simplified Acute Physiology Score II; SOFA, the Sequential Organ Failure Assessment score; BP, blood pressure; SpO_2_, oxyhemoglobin saturation; WBC, white blood cell count; BUN, blood urea nitrogen; INR, international normalized ratio; PTT, partial thromboplastin time; MV, mechanical ventilation; RRT, renal replacement therapy; AKI, acute kidney injury.

### Data extraction and variables processing

In order to maximize the collection of potential candidate delirium predictors, we conducted a comprehensive literature review to summarize the risk factors for delirium. According to the widely accepted classification of risk factors for delirium, these factors can be categorized into three major groups: predisposing factors, disease-related factors, and iatrogenic and environmental factors (6, 29). Old age, gender, body mass index, marital status, education level, and a high burden of coexisting conditions are common predisposing factors (1, 6, 30, 31). The presence of certain chronic comorbidities, such as chronic obstructive pulmonary disease (COPD), hypertension, diabetes, heart failure, atrial fibrillation (AF), stroke, chronic kidney disease (CKD), and tumor has also been associated with the development of delirium (6, 29, 32). The disease-related factors encompass the severity of the disease upon admission and laboratory indicators after admission, including blood routine count, creatinine, electrolyte, albumin, blood glucose, and coagulation indicators (30, 32, 33). The vital signs, including blood pressure, heart rate, respiratory rate, and temperature, are commonly reported as well (34, 35). The iatrogenic and environmental factors involve interventions received in ICUs, including drugs and organ support techniques, such as the utilization of sedatives and vasoactive drugs, and implementation of MV and renal replace therapy (RRT) (6, 29, 36).

Based on the aforementioned delirium-related variables, we utilized structured query language (SQL) with PostgreSQL (version 9.6) to extract the following data from the MIMV-IV v2.2 database: demographic characteristics (including age, gender, race, and marital status), admission condition (including admission type and ICU type), chronic comorbidities, disease severity scores, vital signs and laboratory indicators within 24 h after ICU admission. The vital signs were determined as the mean values during the first 24 h since ICU admission of each included patients. In cases where a laboratory variable was recorded multiple times within this time frame, the value corresponding to the greatest severity of illness was selected. Additionally, we documented the occurrence of acute kidney injury and ICU interventions within 48 h of ICU admission, such as MV, RRT, vasopressors, and sedation.

Our study was retrospective and relied on existing clinical data, no formal sample size calculation was performed prior to the study. Instead, we collected as many samples from the database as possible. Ultimately, a total of 9,748 patients were enrolled in the study. And 48 variables were collected for preliminary analysis (Table 1). Given that this study focuses on a binary outcome, the sample size of the final cohort is adequate to ensure the robustness of the results while adhering to the principle of having at least 10 events per variable (EPV) (37, 38). Variables with missing data exceeding 20% were excluded (39). The remaining missing values underwent multiple imputation using “MICE” package in R (40). Details of missing data was shown in Supplementary Figure S1.

### Statistical analyses

Continuous variables in this study were reported as medians with interquartile range (IQR) unless otherwise specified, and the differences between groups were identified with univariate analysis. Categorical variables were presented as frequency and proportion in each patient group, and compared using the chi-square test or Fisher’s exact test if appropriate. All statistical analyses were performed using the R software (version 4.3.2). *p*-values less than 0.05 (two-sided test) were considered statistically significant.

A pre-seeded random number generator (123) in R software was utilized to randomly divide the cohort into training (*n* = 6,823) and validation (*n* = 2,925) sets based on a ratio of 7:3. All patients in the training set were included for variables selection and model development. We employed an L1-penalty least absolute shrinkage and selection operator (LASSO) regression approach to reduce potential collinearities and prevent overfitting, augmented with 10-fold cross-validation (41). LASSO regression is a method used to reduce the dimensionality of data by selecting features based on a penalty function. It effectively reduces the absolute size of the coefficients in a regression model, determined by the value of lambda. Following the feature selection, we identified 26 features with significant predictive ability according to lambda. 1se criterion. The prediction model was then constructed using the following ML algorithm, including logistic regression (LR), decision trees (DT), support vector machines (SVM), extreme gradient boosting (XGBoost), k-nearest neighbors (KNN), and naive Bayes (NB). ML have the capacity to accommodate numerous predictors, fewer model assumptions, and require less user specification of model terms. It has the ability to form flexible, empirically driven interactions based on the data without needing these interactions to be specified in advance (20). During the modeling process, we repeated 5 rounds of 10-fold cross-validation and grid search parameter optimization to ensure stability.

The area under receiver operating characteristic (ROC) curve (AUC), accuracy, specificity, sensitivity, positive predictive value (PPV), negative predictive value (NPV), recall, and F1 score were used to assess the model’s performance. The optimal model was determined based on the highest AUC and accuracy in the validation set (42, 43). We then utilized a calibration curve to evaluate the consistency between predicted and actual occurrence of delirium for the top three optimal models in the training set. Additionally, we assessed the net clinical benefit through the decision curve analysis (DCA).

SHAP method is applied to interpret the optimal model. The SHAP values are derived from game theory, providing an estimation of the impact that each feature has on the predicted outcome and effectively explaining the contribution of each feature to a single observation (19, 44). We employed a SHAP significance analysis and SHAP summary plot to evaluate feature importance, followed by utilizing SHAP dependency plot to investigate the impact of features on outcome prediction. Finally, a SHAP force analysis was used to elucidate the contribution of features in individual patients.

## Results

### Baseline characteristics

**Figure 1:**
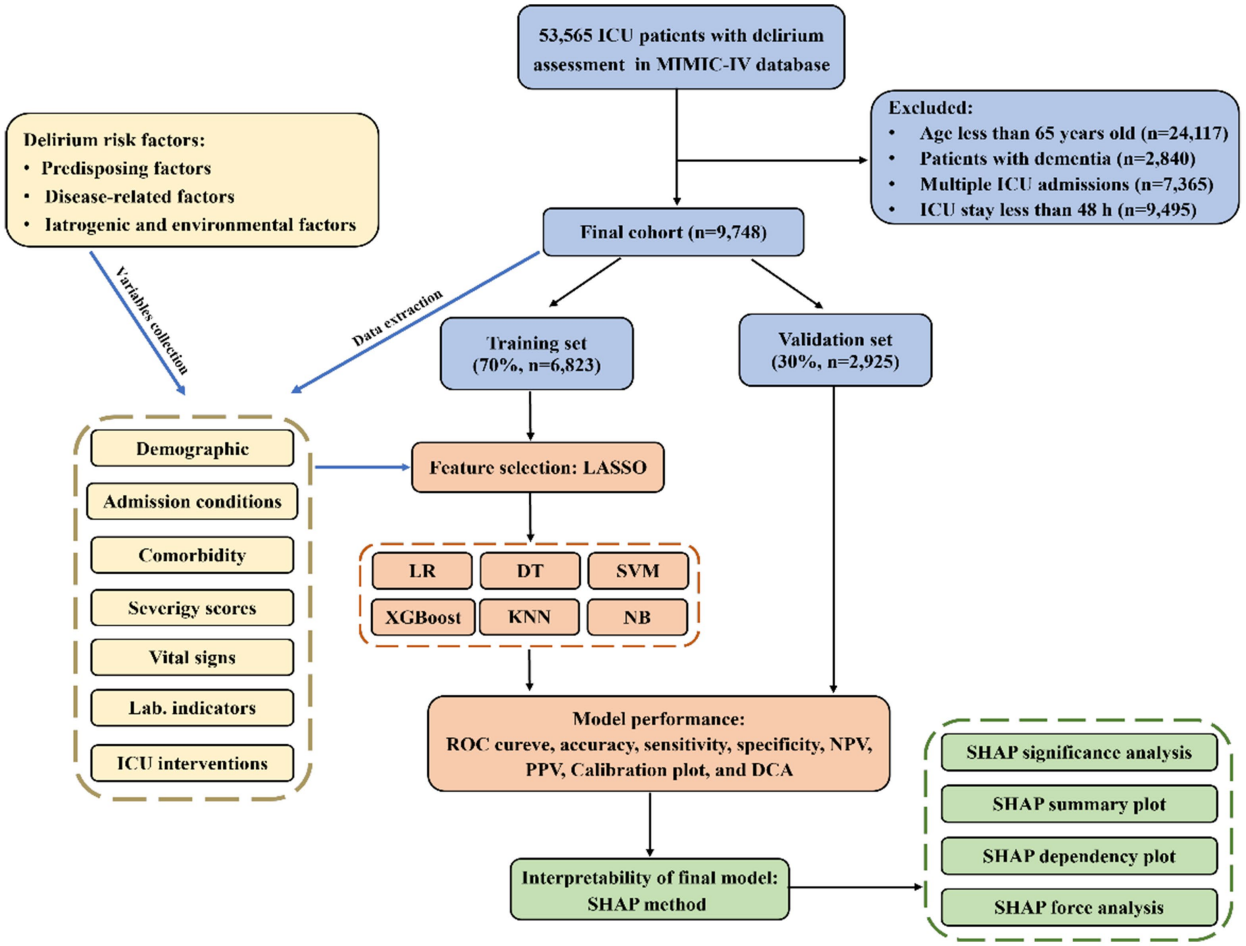
The flowchart and framework of the prediction models.

A total of 9,748 older patients from the MIMIC-IV v2.2 database were eventually included in this study and the detailed selection process could be found in Figure 1. Among the enrolled patients, there were 4,243 cases of delirium (43.5%). Table 1 summarizes the characteristics of patients with and without delirium, including the demographic, comorbidity, disease-related conditions, and the ICU interventions. Overall, patients with delirium had had higher white blood cell, blood urea nitrogen, creatinine, anion gap, international normalized ratio and glucose levels, and were more likely to have COPD, cerebrovascular disease, diabetes, CKD, and stroke, and received more medical treatment. They also exhibited more abnormal vital signs and electrolyte levels, as well as a higher degree of disease severity. The length of the ICU and hospital day in the delirium group was significantly longer than that in the non-delirium group [ICU-stay: 5.3 (3.3, 9.5) vs. 3.1 (2.3, 4.2), *p* < 0.001; hospital-stay: 12 (8, 20) vs. 8 (5, 12), *p* < 0.001]. Similarly, there were significant difference in mortality between delirium and non-delirium groups (ICU mortality: 13.0% vs. 5.7%, *p* < 0.001; hospital mortality: 22.0% vs. 10.0%, *p* < 0.001), which suggests that delirium may be associated with a poor prognosis.

The total population was divided into a 70% training cohort and a 30% validation cohort, with comparable baseline characteristics between the two sets (*p* > 0.05), as detailed in Supplementary Table S1. The training set was subsequently utilized for model development.

### Feature selection and model development

**Figure 2:**
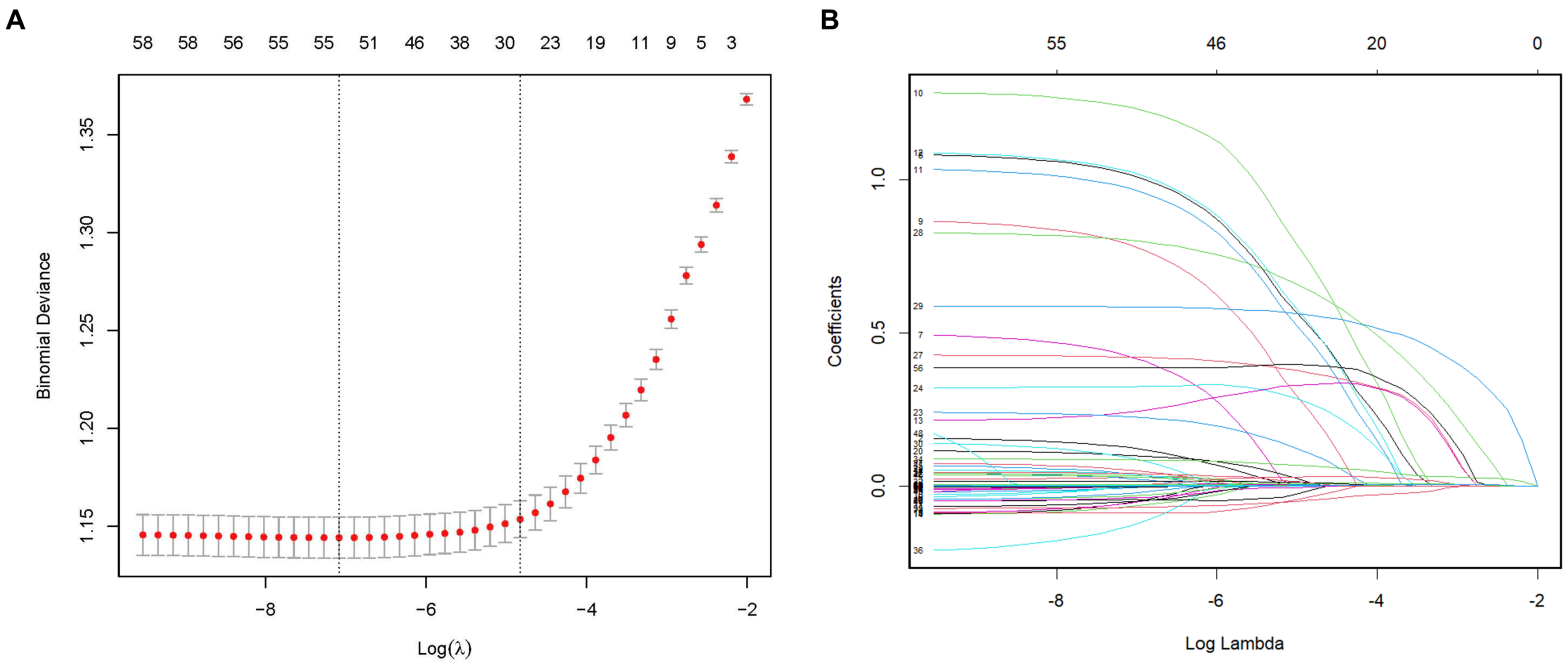
Feature selection by the LASSO regression model. **(A)** The LASSO model underwent tenfold cross-validation to determine the optimal penalization coefficient parameter (lambda). **(B)** The plots depict the LASSO regression coefficients across various penalty parameter values. The lambda. 1se was chosen in our study due to its stricter penalty and ability to reduce overfitting. LASSO, least absolute shrinkage and selection operator.

To identify the most relevant variables for critical delirium in Table 1, we employed L1-penalized LASSO regression for dimensionality reduction and feature selection. Figure 2A illustrates the relationship between cross-validation errors and penalty terms. We utilized a 10-fold cross-validation approach to determine the optimal penalty parameter lambda, selecting 26 clinical variables with significant predictive ability based on the lambda. 1se criteria to construct our model. Figure 2B displays the distribution of coefficients for these selected features in the LASSO regression, revealing the optimal point for retaining nonzero variables. The Supplementary Table S2 presents the 26 selected variables, along with their corresponding non-zero coefficient values.

Subsequently, based on the selected features, we employed six ML algorithms, including LR, DT, SVM, XGBoost, KNN, and NB, to predict the primary outcome from the training set. During the modeling process, we performed 5 rounds of 10-fold cross-validation and grid search parameter optimization to ensure the generalizability of the models while avoiding overfitting.

### Model performance and comparisons

**Figure 3:**
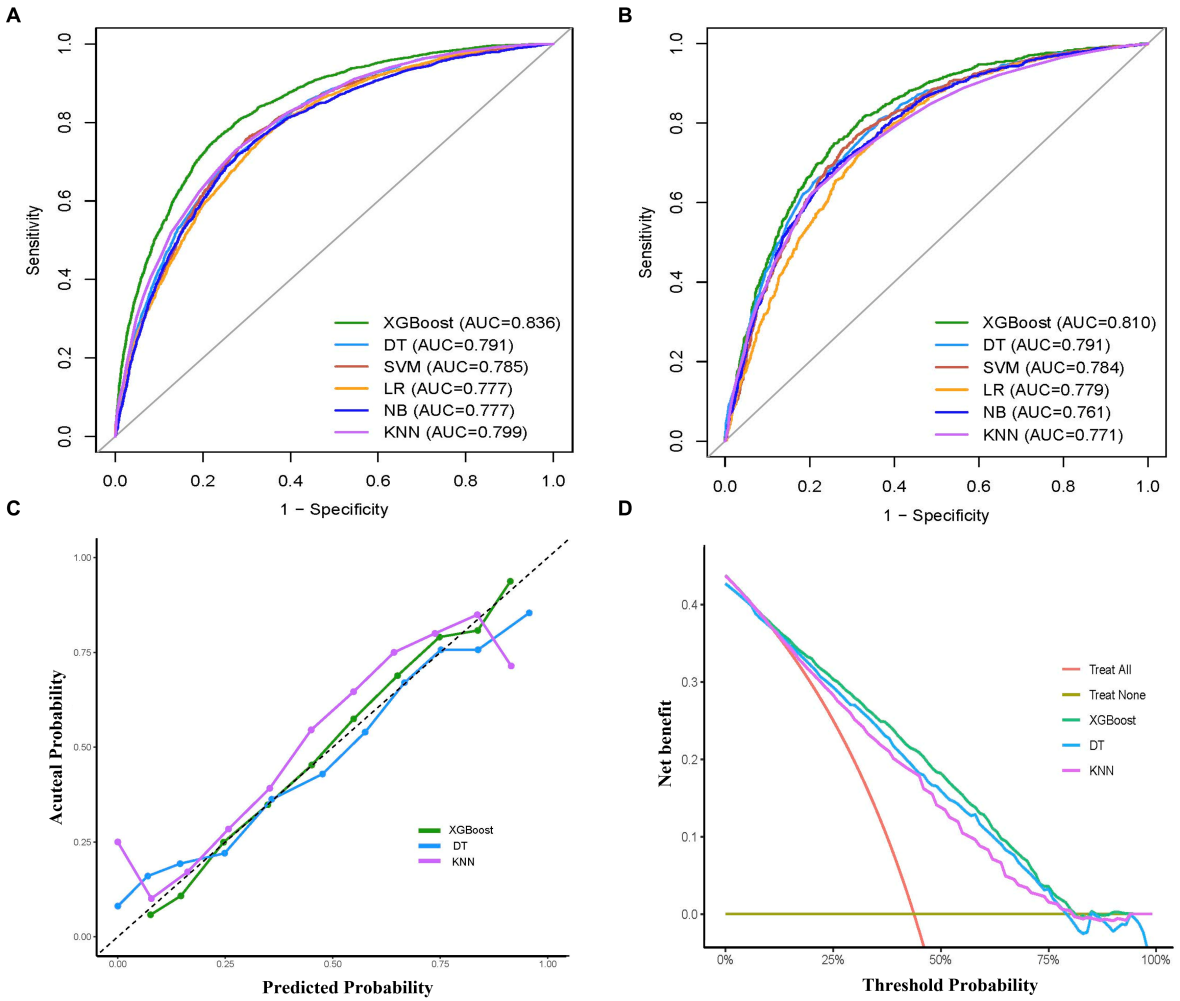
Comprehensive evaluation of machine learning models. **(A)** ROC curves and AUC values of the training set. **(B)** ROC curves and AUC values of the validation set. **(C)** Calibration curves of the XGBoost, DT, KNN models in the validation set. **(D)** Decision curves analysis of the XGBoost, RF, SVM models in the validation set. ROC, receiver operating characteristic; AUC, the area under the receiver operating characteristic curve; LR, logistic regression; XGBoost, extreme gradient boosting; DT, decision tree; SVM, support vector machine; KNN, k-nearest neighbors; NB, naive bayes.

The performance comparison of various ML models was presented in Table 2 and Figure 3, respectively. Table 2 provides the detailed AUC, accuracy, sensitivity, specificity, PPV, NPV, recall, and F1 scores for six models. The AUC values associated with the different models ranged from 0.777 to 0.836 (LR: 0.777, DT: 0.791, SVM: 0.785, XGBoost: 0.836, KNN: 0.799, and NB: 0.777) in the training set (Figure 3A). The XGBoost model had the highest performance with an AUC of 0.836, accuracy of 0.765, sensitivity of 0.713, recall of 0.713, and F1 score of 0.725 (Table 2). Similarly, in the validation set, the XGBoost model achieved the highest performance with an AUC of 0.810 and accuracy of 0.744, which surpassed the AUCs of the other models, highlighting the superior performance of the XGBoost model (Table 2 and Figure 3B).

**Table 2:** The prediction performance of each model.

Model	AUC	Accuracy	Sensitivity	Specificity	PPV	NPV	Recall	F1 score
*Training set*								
LR	0.777	0.713	0.599	0.801	0.698	0.722	0.599	0.645
XGBoost	0.836	0.765	0.713	0.804	0.737	0.785	0.713	0.725
DT	0.791	0.724	0.683	0.755	0.682	0.756	0.683	0.683
SVM	0.785	0.721	0.636	0.787	0.696	0.738	0.636	0.665
KNN	0.799	0.719	0.519	0.873	0.758	0.703	0.519	0.616
NB	0.777	0.678	0.399	0.892	0.739	0.659	0.399	0.518
*Validation set*								
LR	0.780	0.715	0.602	0.804	0.704	0.722	0.602	0.649
XGBoost	0.810	0.744	0.692	0.785	0.715	0.766	0.692	0.703
DT	0.792	0.722	0.671	0.761	0.686	0.748	0.671	0.679
SVM	0.785	0.720	0.638	0.785	0.697	0.736	0.638	0.666
KNN	0.772	0.700	0.498	0.858	0.731	0.687	0.498	0.592
NB	0.761	0.662	0.385	0.878	0.710	0.647	0.385	0.499

LR, logistic regression; XGBoost, extreme gradient boosting; DT, decision tree; SVM, support vector machine; KNN, k-nearest neighbors; NB, naive bayes.

To examine the calibration of the models, calibration curves for the three models with the highest AUC values (XGBoost, KNN, DT) were generated and compared (Figure 3C). Among them, XGBoost showed the best fit between observed and predicted probabilities, indicating its superior calibration. Decision curve analysis (DCA) was performed on these three models and the results are shown in Figure 3D. The analysis showed that using the XGBoost prediction model provided the highest net benefit for predicting delirium, outperforming both KNN and DT. Taken together, the XGBoost model was selected as the optimal model and subsequently employed for further interpretation.

### Model interpretations

**Figure 4:**
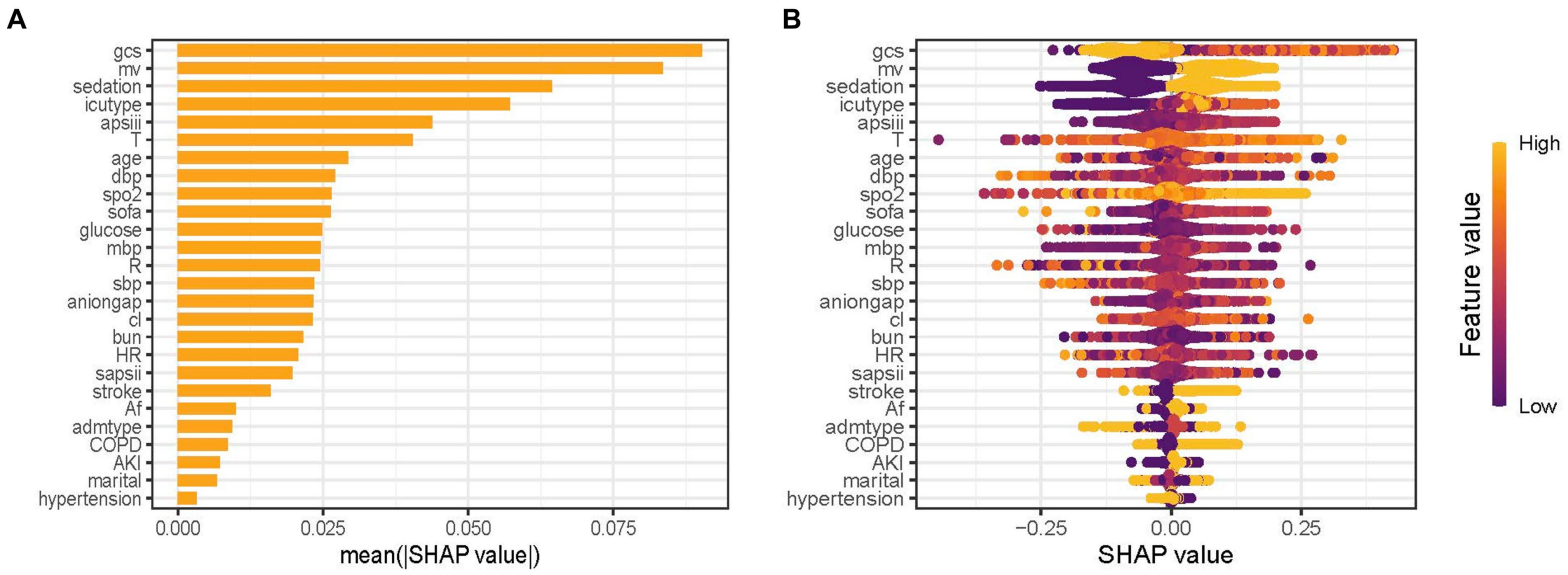
Feature importance analysis by SHAP method for XGBoost model. **(A)** SHAP significance analysis of feature importance ranking based on the mean value. **(B)** SHAP summary plot of the XGBoost model. GCS, Glasgow Coma Scale; MV, mechanical ventilation; APSIII, the Acute Physiology Score III; T, temperature; DBP, diastolic blood pressure; SpO_2_, oxyhemoglobin saturation; SOFA, the Sequential Organ Failure Assessment Score; MBP, mean blood pressure; R, respiratory rate; SBP, systolic blood pressure; Cl, chloride; BUN, blood urea nitrogen; HR, heart rate; SAPSII, the Simplified Acute Physiology Score II; AF, Atrial fibrillation; Admtype, type of admission; COPD, chronic obstructive pulmonary disease; AKI, acute kidney injury.

The predictor’s contribution to the prediction outcomes was quantified using SHAP, which employs a game-theoretic approach to assess the significance of each feature. The feature importance ranking was visualized using the SHAP significance analysis for the XGBoost model, as depicted in Figure 4A. Our analysis identified the top 10 risk factors associated with critical delirium, including Glasgow Coma Scale (GCS) score, MV, sedation, ICU type, the Acute Physiology Score III (APSIII), temperature, age, diastolic blood pressure, oxyhemoglobin saturation and the Sequential Organ Failure Assessment score (SOFA). This ranking was further complemented by SHAP summary plot (Figure 4B) that visually demonstrates the influence of each feature on model output. A positive Shapley value for each feature indicates an increased risk of delirium while a negative value suggests decreased risk. For instance, for MV, yellow dots located rightward from zero line signifies higher MV values (i.e., receiving MV treatment) contributing towards increased risks of delirium.

**Figure 5:**
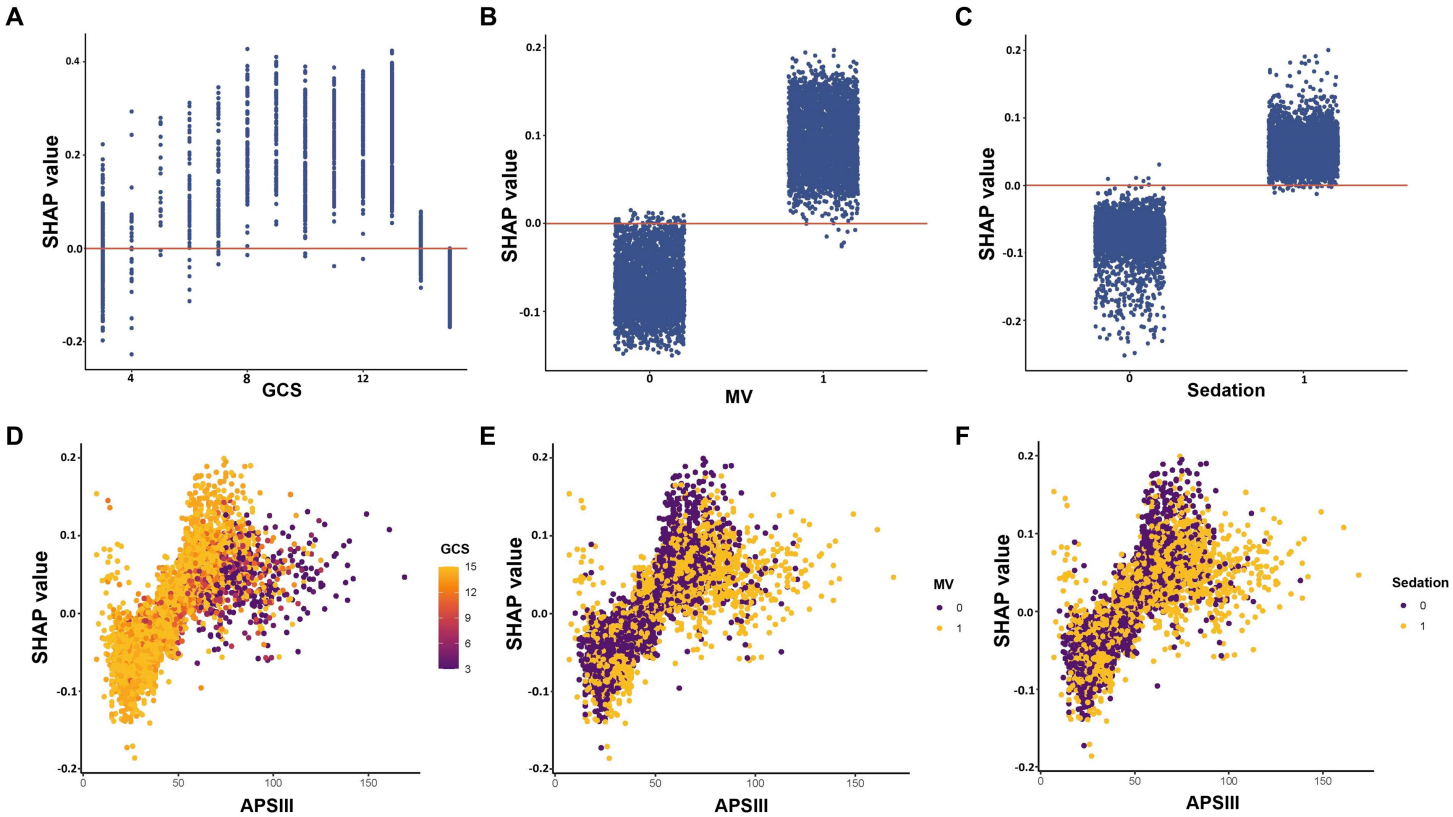
SHAP dependency plot of features in the XGBoost model. The *Y*-axis represents SHAP values, while the *X*-axis represents actual clinical parameters. For binary variables such as MV and sedation, “0” indicates the absence of the condition, while “1” indicates its presence. Significantly, when a feature’s SHAP value is greater than 0, it suggests an increased risk of delirium, whereas a negative SHAP value suggests a reduced risk. GCS, Glasgow Coma Scale; MV, mechanical ventilation; APSIII, the Acute Physiology Score III.

The impact of features at factor level on the risk of the predictive model was analyzed using SHAP dependency plot, as depicted in Figure 5. The three most important features in the XGBoost model, namely GCS, MV, and sedation, were depicted in Figures 5A–C respectively. The results showed a complex nonlinear relationship between GCS and outcomes, while MV and sedation were consistently associated with increased risk. APSIII score is a widely used tool to assess the severity of patients in the ICU. Using the APSIII score as an example, Figures 5D–F furthermore illustrated interactions among different features. It was evident that despite identical APSIII scores, there may be discrepancies in the corresponding SHAP values for different levels of GCS, MV and sedation.

Additionally, we further demonstrate the model’s interpretability by presenting SHAP force analysis for two representative cases: one predicting a high risk of delirium and another indicating a low risk of delirium (Supplementary Figure S2). The plot provides an overview of how the key features affect prediction outcome at individual level. Factors that contribute to higher predicted scores compared with the baseline (mean predicted value) are highlighted in purple, while factors that lead to lower predicted scores are indicated in orange. The length of the arrows helps visualize the degree of impact of the prediction, whereby the longer the arrow, the more significant the effect. For instance, in the first case (Supplementary Figure S2A), most features are shown in purple, suggesting their contribution to the risk of developing delirium, particularly blood urea nitrogen and APSIII.

## Discussion

In this retrospective cohort study, we used ML methods to establish a clinical prediction model for assessing the risk of delirium in ICU patients aged 65 years and older. The ML prediction model based on XGBoost was ultimately chosen due to its impressive performance in predicting delirium. In addition, we further used the SHAP value method to gain a deeper understanding of the prediction model. To the best of our knowledge, this study is the first to develop a prediction model for delirium in older patients in the ICU through explainable ML methods. These findings could help healthcare providers identify delirium early in daily clinical practice and assist in medical decision-making.

Delirium is the most common neuropsychological complication during ICU stay for older patients. Delirium among older patients could lead to prolonged hospitalization day, increased mortality, and diminished long-term quality of life (5, 6, 8). Early recognition of risk factors related to delirium is important. The establishment of reliable delirium prediction models could assist clinicians in identifying high-risk patients and guiding timely intervention. Although several models have been developed to assess the risk of delirium in ICU, these models either encompass a wide range of age groups or solely focus on the recovery period after surgery, without considering the specific characteristics of older patients in ICU settings (45–47). As far as we know, this is the first study on the risk prediction of delirium in critically ill patients aged 65 years and older. The best ML model selected in this study, namely XGBoost, showed good discrimination, calibration and clinical practicability in predicting the risk of delirium in ICU older patients. Recently, Marra et al. (14) developed a dynamic model to predict the risk of delirium in ICU patients. The model had a high negative predictive value (0.874) in excluding the next-day delirium, but a poor positive predictive value (0.548) and sensitivity (0.597). This suggests that the model is mainly used to exclude the risk of delirium, rather than identify high-risk patients (45). In contrast, our model not only has a high AUC value and accuracy, but also has good specificity, sensitivity, PPV, and NPV in both the training and validation sets. Therefore, it has higher clinical value in guiding targeted interventions to prevent older delirium in ICUs.

Feature selection is a crucial step in developing prediction models (48). Based on an extensive review of previously published literature on delirium risk factors, we have identified potential predictors of delirium and then comprehensively screened these risk factors from the database. It is noteworthy that we obtained a substantial sample size from the MIMIC-IV database, enabling us to incorporate a greater number of potential risk factors in our feature selection (37). This is crucial for identifying important predictive variables. We then utilized the LASSO regression to feature processing, which can avoid model overfitting and exclude the influence of strong collinearity related variables (49). In addition, the utilization of ML techniques to build prediction models can also easily handle multiple variables and capture nonlinear relationships (21). In the past, several studies have developed prediction models for delirium in the ICU. The PRE-DELIRIC and early PRE-DELIRIC model includes predictive variables such as age, illness severity score, patient classification, coma, use of sedatives and analgesics, and emergency admission; while the Lanzhou model incorporates mechanical ventilation, coma, blood urea nitrogen and mean arterial pressure at ICU admission, and medical history as predictive variables (12, 13, 15, 50). However, these models are built on traditional regression analysis methods with limited inclusion of population and candidate variables. They also target a broader age group and cannot reflect the specific characteristics of older patients. Our study focused on older ICU patients, as they are more to suffer from delirium (3). We extensively screened potential risk factors associated with critical delirium in older adults. We also found that the advanced age, severity score, use of sedation, type of admission and type of ICU, BUN, and mean BP was associated with the occurrence of delirium in older adults. In addition to these aforementioned risk factors, certain vital signs such as temperature, heart rate, respiratory rate, and SpO_2_ also hold predictive value in our findings. These vital signs also reflect the severity of illness in critically ill older patients. Previous research has indicated that a history of conditions such as hypertension, chronic obstructive pulmonary disease, and diabetes is linked to the occurrence of delirium (6, 29). However, our findings suggest that certain comorbidities, including acute kidney injury, stroke, and atrial fibrillation, have a higher predictive value for the risk of delirium in older individuals. It is worth noting that the analysis results also found that marital status impacts delirium occurrence: married older patients had a lower risk of delirium in the prediction model. This aspect has received less attention in previous studies on non-older patients, possibly because marital status affects the emotional state of older patients, which in turn influences delirium occurrence (51, 52). Further research is needed to confirm this hypothesis.

The interpretability of ML has always been a challenging problem (18). To address this issue, we employed the SHAP values to analyze each feature and enhance the interpretability of the model (19). Based on the SHAP importance ranking, it is visually evident that the important features significantly influence the occurrence of delirium in older patients within ICUs. Notably, advanced age, low GCS score, high SOFA score, high APSIII score, MV treatment, and sedative use have all been widely reported as risk factors for delirium (6, 29, 31, 32). Recently, Zhang et al. (53) used ML methods to develop a prediction model for patients with sepsis-related delirium. The model successfully identified the top 10 important features impacting outcomes, including MV, initial ICU type, GCS, sedation, temperature, and age. This has high consistency with the predictive features obtained in our study. However, due to different study outcomes, there are discrepancies in the ranking of feature importance. Interestingly, we observed that there is a complex nonlinear correlation between GCS and the predicted outcome through the SHAP dependency plot, which has also been observed in other delirium prediction models (53). From a clinical perspective, a GCS score of 3 indicates severe brain damage, while a score of 15 suggests normal brain function. Therefore, patients in both groups had significantly reduced risk of developing delirium. Additionally, the use of SHAP force plots also provides personalized prediction insights for delirium, visually guiding clinicians and patients in decision-making. Taken together, the combination of XGBoost and SHAP can provide clear explanations for personalized risk prediction, facilitating an enhanced comprehension of the efficacy of important features within the model.

There are several limitations in this study. Firstly, not all patients in the database received CAM-ICU evaluation for delirium diagnosis, and this study excluded those who did not receive delirium assessment, which may lead to selection bias in the sample population. Secondly, despite our best efforts to collect potential predictors of delirium, some risk factors such as education level, alcohol consumption history, and activities of daily living were not recorded in the database, so we were unable to obtain this information. In fact, these factors may also have an impact on the occurrence of delirium after admission (29, 36). Also, several variables had to be excluded due to a high number of missing values. These may have caused us to overlook some features. Thirdly, we could not conduct further analysis on the potential effects of MV duration, types and doses of sedative drugs used in older adults within the ICU, which may potentially complicate our predictive variables for older delirium. Finally, the model has been validated and demonstrated excellent performance in the internal validation cohorts; however, it lacks external validation. While ML has the potential to improve clinical care by providing prediction for the risk of delirium in older adults, researchers should critically evaluate data sources, feature selection, and machine learning algorithms (20). In clinical practice, researchers should use an analysis framework that is consistent with the research objectives of this study, and conduct prospective cohort studies to verify the generalizability and reproducibility of results. Interdisciplinary research teams, including machine learning experts and clinical specialists, should work together to validate and evaluate prediction models. The interpretation of predictive outcomes should be more closely integrated with clinical practice in order to better improve patient care.

## Conclusion

In summary, our study developed a ML model based on the MIMIC-IV v2.2 databases for early prediction of delirium risk in older ICU patients. The XGBoost model outperformed other models in terms of prediction performance. The SHAP methods were used to explain intrinsic information of the XGBoost model, which can provide clear explanations for personalized risk prediction and facilitate a more intuitive understanding of the effects of key features. These findings have the potential to assist clinicians in screening older patients at high risk of critical delirium and help optimize management strategies.

## Data availability statement

Publicly available datasets were analyzed in this study. This data can be found here: https://mimic.mit.edu.

## Ethics statement

The studies involving humans were approved by the Massachusetts Institute of Technology and the Beth Israel Deaconess Medical Center. The studies were conducted in accordance with the local legislation and institutional requirements. The participants provided their written informed consent to participate in this study. Written informed consent was obtained from the individual(s) for the publication of any potentially identifiable images or data included in this article.

## Author contributions

DT: Data curation, Formal analysis, Investigation, Methodology, Software, Validation, Writing – original draft. CM: Conceptualization, Methodology, Project administration, Resources, Supervision, Writing – review & editing. YX: Conceptualization, Investigation, Methodology, Project administration, Supervision, Writing – review & editing.
