# Supplementary material for: Interpretable machine learning model for early prediction of delirium in elderly patients following intensive care unit admission: a derivation and validation study
Source: Front Med (Lausanne). 2024 May 17;11:1399848. doi: 10.3389/fmed.2024.1399848 (PMC11140063; doi:10.3389/fmed.2024.1399848)
Supplement: Supplementary file 1 [file Data_Sheet_1.docx]

**Interpretable machine learning model for early prediction of delirium in elderly patients following intensive care unit admission: a derivation and validation study**

Dayu Tang^1^, Chengyong Ma^1*^, Yu Xu^1*^

^1^ Department of Critical Care Medicine, West China Hospital, Sichuan University, Chengdu, China.

* Correspondence: yongdoctorma@wchscu.cn(CY.M.); xuyu14167@163.com(Y.X.)

Supplementary Materials


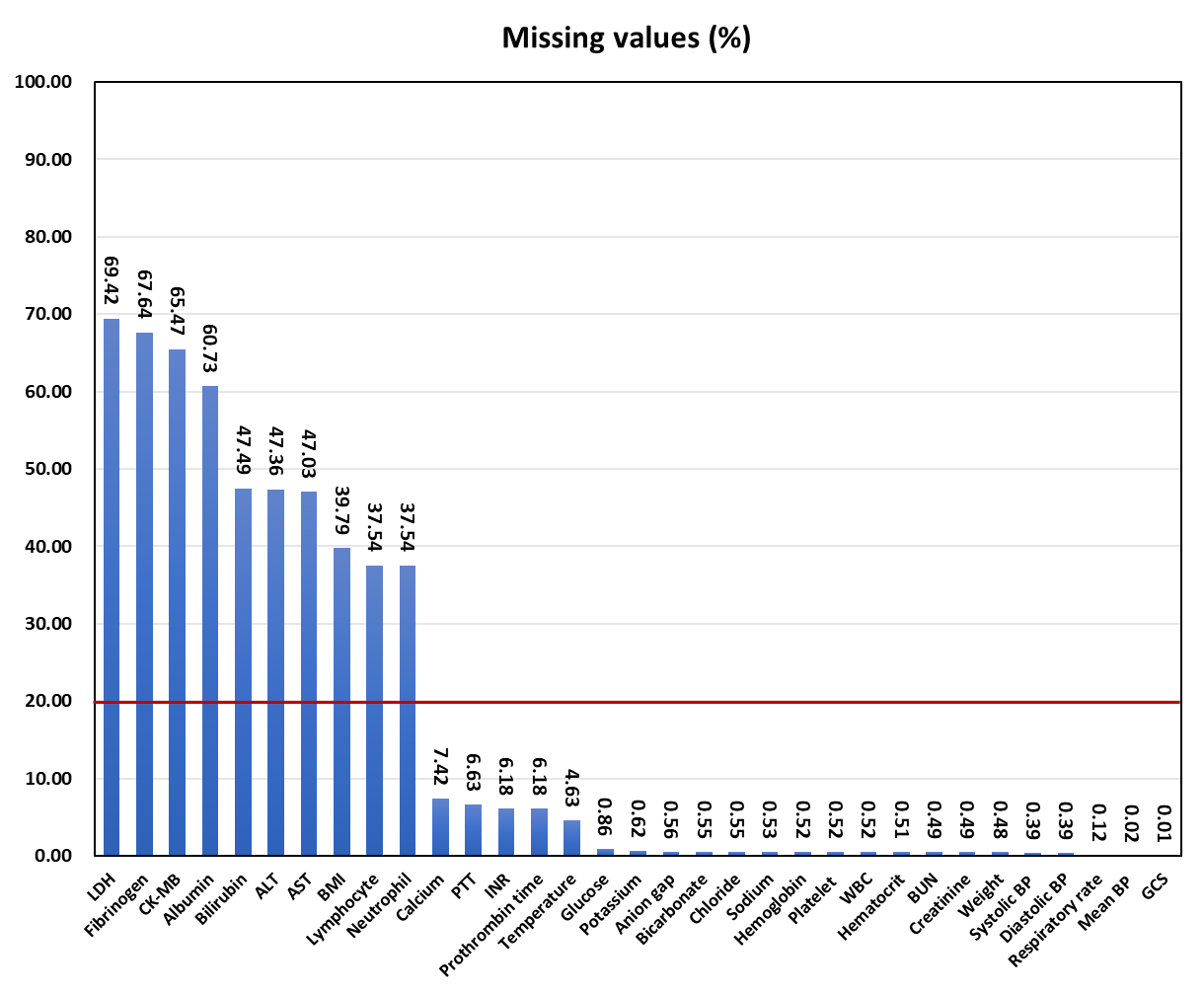


**Fig. S1** The percentage of missing values (%) for variables in the dataset. Notably, we removed variables missing > 20% of observations to facilitate and ensure study accuracy. *LDH* lactic dehydrogenase, *CK-MB* creatine kinase-MB, *ALT* alanine transaminase, *AST* alanine transaminase, *BMI* body mass index, *PTT* partial thromboplastin time, *INR* international normalized ratio, *WBC* white blood cell, *BUN* blood urea nitrogen, *BP* blood pressure, *GCS* Glasgow Coma Score.

**Table S1** Baseline characteristics in the training and testing sets

| **Variables** | **Training set**  **(N=6,823)** | **Validation set**  **(N=2,925)** | **P value** |
| --- | --- | --- | --- |
| Age (years) | 76 (70, 83) | 76 (70, 83) | >0.9 |
| Male (%) | 3,707 (54.3%) | 1,570 (53.7%) | 0.6 |
| Ethnicity (%) |  |  | 0.4 |
| Asian | 1,189 (17.4%) | 499 (17.1%) |  |
| Black | 514 (7.5%) | 251 (8.6%) |  |
| Hispanic | 144 (2.1%) | 66 (2.3%) |  |
| White | 4,689 (68.7%) | 1,976 (67.6%) |  |
| Others | 287 (4.2%) | 133 (4.5%) |  |
| Marital Status (%) |  |  | 0.8 |
| Single | 1,717 (25.2%) | 759 (25.9%) |  |
| Married | 3,341 (49.0%) | 1,430 (48.9%) |  |
| Divorced | 464 (6.8%) | 194 (6.6%) |  |
| Others | 1,301 (19.1%) | 542 (18.5%) |  |
| Admission type (%) |  |  | 0.2 |
| Selective | 1,108 (16.2%) | 430 (14.7%) |  |
| Urgent | 5,467 (80.1%) | 2,383 (81.5%) |  |
| Emergent | 248 (3.6%) | 112 (3.8%) |  |
| ICU type (%) |  |  | 0.6 |
| CVICU | 1,579 (23.1%) | 628 (21.5%) |  |
| CCU | 977 (14.3%) | 428 (14.6%) |  |
| MICU | 1,022 (15.0%) | 435 (14.9%) | 0.2 |
| M/SICU | 914 (13.4%) | 403 (13.8%) |  |
| NICU | 739 (10.8%) | 311 (10.6%) |  |
| SICU | 881 (12.9%) | 405 (13.8%) |  |
| TSICU | 711 (10.4%) | 315 (10.8%) | 0.8 |
| Comorbidity |  |  |  |
| COPD (%) | 795 (11.7%) | 338 (11.6%) | 0.9 |
| Hypertension (%) | 3,233 (47.4%) | 1,405 (48.0%) | 0.6 |
| Diabetes (%) | 2,289 (33.5%) | 949 (32.4%) | 0.3 |
| Heart failure (%) | 2,588 (37.9%) | 1,118 (38.2%) | 0.8 |
| Atrial fibrillation (%) | 3,097 (45.4%) | 1,300 (44.4%) | 0.4 |
| AMI (%) | 1,016 (14.9%) | 432 (14.8%) | 0.9 |
| CKD (%) | 1,705 (25.0%) | 714 (24.4%) | 0.5 |
| Stroke (%) | 1,468 (21.5%) | 644 (22.0%) | 0.6 |
| Tumor (%) | 1,007 (14.8%) | 476 (16.3%) | 0.056 |
| Scoring system |  |  |  |
| GCS | 15.0 (14.0,15.0) | 15.0 (14.0,15.0) | 0.3 |
| APSIII | 43 (33, 56) | 43 (33, 56) | 0.4 |
| SAPS II | 39 (32, 48) | 39 (32, 48) | 0.5 |
| SOFA | 5.0 (3.0, 7.0) | 5.0 (3.0, 7.0) | 0.6 |
| Vital Signs |  |  |  |
| Heart rate (min^-1^) | 81 (73, 93) | 81 (72, 92) | 0.5 |
| Systolic BP (mmHg) | 116 (107, 128) | 116 (106, 128) | 0.7 |
| Diastolic BP (mmHg) | 59 (53, 67) | 59 (53, 67) | >0.9 |
| Mean BP (mmHg) | 75 (70, 83) | 75 (70, 83) | >0.9 |
| Respiratory rate (min^-1^) | 18.8 (16.8, 21.4) | 18.7 (16.7, 21.2) | 0.1 |
| Temperature (℃) | 36.80(36.60,37.05) | 36.79 (36.60,37.04) | 0.4 |
| SpO_2_(%) | 97.08(95.63,98.42) | 97.17 (95.65,98.48) | 0.14 |
| Lab. indicators |  |  |  |
| WBC (10^9^/L) | 11.3 (8.4, 14.9) | 11.3 (8.3, 15.2) | 0.8 |
| Hemoglobin (10^12^/L) | 10.45 (9.13, 12.00) | 10.50 (9.20, 12.00) | 0.4 |
| Hematocrit (%) | 32.0 (28.1, 36.6) | 32.0 (28.2, 36.8) | 0.5 |
| Platelet (10^9^/L) | 180 (135, 240) | 180 (135, 238) | 0.7 |
| Bicarbonate(mmol/L) | 23.0 (20.5, 25.0) | 23.0 (20.5, 25.0) | 0.6 |
| ***Table 1 (continued)*** |  |  |  |
| **Variables** | **Training set**  **(N=6,823)** | **Validation set**  **(N=2,925)** | **P value** |
| Sodium (mmol/L) | 138.5(136.0,141.0) | 138.5 (136.0,141.0) | 0.6 |
| Potassium(mmol/L) | 4.20 (3.90, 4.60) | 4.20 (3.90, 4.60) | 0.2 |
| Chloride (mmol/L) | 104.0(100.0,107.5) | 104.0 (100.0, 07.0) | 0.7 |
| Calcium (mmol/L) | 8.40 (7.90, 8.85) | 8.40 (7.95, 8.85) | 0.3 |
| Glucose (mg/dL) | 132 (111, 163) | 132 (112, 164) | 0.9 |
| BUN (mg/dL) | 22 (16, 34) | 22 (15, 35) | 0.9 |
| Creatinine (mg/dL) | 1.05 (0.80, 1.55) | 1.05 (0.80, 1.55) | 0.6 |
| Anion gap (mmol/L) | 14.5 (12.5, 17.0) | 14.5 (12.5, 17.0) | 0.5 |
| INR | 1.25 (1.10, 1.50) | 1.25 (1.10, 1.50) | 0.8 |
| Prothrombin time (s) | 13.8 (12.2, 16.4) | 13.7 (12.3, 16.2) | 0.8 |
| PTT (s) | 32 (28, 42) | 32 (28, 42) | >0.9 |
| ICU interventions |  |  |  |
| MV (%) | 3,176 (46.5%) | 1,341 (45.8%) | 0.5 |
| RRT (%) | 301 (4.4%) | 119 (4.1%) | 0.4 |
| Vasopressor use (%) | 3,199 (46.9%) | 1,323 (45.2%) | 0.13 |
| Sedation (%) | 3,672 (53.8%) | 1,567 (53.6%) | 0.8 |
| AKI (%) | 4,894 (71.7%) | 2,113 (72.2%) | 0.6 |
| ICU-stay (days) | 3.7 (2.6, 6.1) | 3.7 (2.7, 6.0) | 0.9 |
| Hospital-stay (days) | 9 (6, 15) | 9 (6, 15) | >0.9 |
| ICU-mortality (%) | 604 (8.9%) | 261 (8.9%) | >0.9 |
| Hospital-mortality (%) | 1,043 (15%) | 443 (15%) | 0.9 |

Data are presented as a number with the percentage in parentheses, or as the median with the interquartile range in parentheses. The "tumor" refers to a malignant cancer. Sedation includes midazolam, propofol, dexmedetomidine, and diazepam. *ICU* intensive care unit, *CCU* coronary care unit, *CVICU* cardiovascular ICU, *MICU* medical ICU, *SICU* surgical ICU, *NICU* neuro ICU, *TSICU* trauma-neuro surgical ICU, *COPD* chronic obstructive pulmonary disease, *AMI* acute myocardial infarction, *CKD* chronic kidney disease, *GCS* Glasgow Coma Score, *APSIII* the Acute Physiology Score III, *SAPS II* the Simplified Acute Physiology Score II, *SOFA* the Sequential Organ Failure Assessment score, *BP* blood pressure, *SpO_2_* oxyhemoglobin saturation, *WBC* white blood cell count, *BUN* blood urea nitrogen, *INR* international normalized ratio, *PTT* partial thromboplastin time, *MV* mechanical ventilation, *RRT* renal replacement therapy, *AKI* acute kidney injury.

**Table S2** Variables selected by LASSO regression and coefficients of variables (β)

| **Variables** | **Coefficient (β)** | **Variables** | **Coefficient (β)** |
| --- | --- | --- | --- |
| Intercept | -0.1946728 | SAPSII | 0.001951562 |
| Age | 0.01157584 | COPD | 0.2688934 |
| Temperature | 0.3950748 | Hypertension | -0.01408490 |
| Heart Rate | 0.0009718893 | Atrial fibrillation | 0.01173537 |
| Respiratory Rate | 0.002293311 | Stroke | 0.3701769 |
| SpO_2_ | 0.01052727 | AKI | 0.1061685 |
| Diastolic BP | 0.004211937 | Sedation | 0.6370383 |
| Systolic BP | 0.002709297 | MV | 0.5587441 |
| Mean BP | 0.0001422814 | Admission type: urgent | 0.3327186 |
| BUN | 0.0004618831 | Marital status: married | -0.03447364 |
| Chloride | -0.002568728 | ICU type: MICU | 0.5174732 |
| Glucose | 0.001668954 | ICU type: M/SICU | 0.2392850 |
| Anion gap | 0.03100068 | ICU type: NICU | 0.7248545 |
| GCS | -0.04559880 | ICU type: SICU | 0.4711153 |
| SOFA | 0.06608223 | ICU type: TSICU | 0.5245321 |
| APSIII | 0.008688745 |  |  |

These coefficients are calculated at a lambda.1se value, which is selected by tenfold cross-validation to minimize the sum of squared errors of the model. Sedation includes midazolam, propofol, dexmedetomidine, and diazepam. *SpO_2_* oxyhemoglobin saturation, *BP* blood pressure, *BUN* blood urea nitrogen, *GCS* Glasgow Coma Score, *APSIII* the Acute Physiology Score III, *SAPS II* the Simplified Acute Physiology Score II, *COPD* chronic obstructive pulmonary disease, *AKI* acute kidney injury, *MV* mechanical ventilation, *ICU* intensive care unit, *M/SICU* medical and surgical ICU, *SICU* surgical ICU.


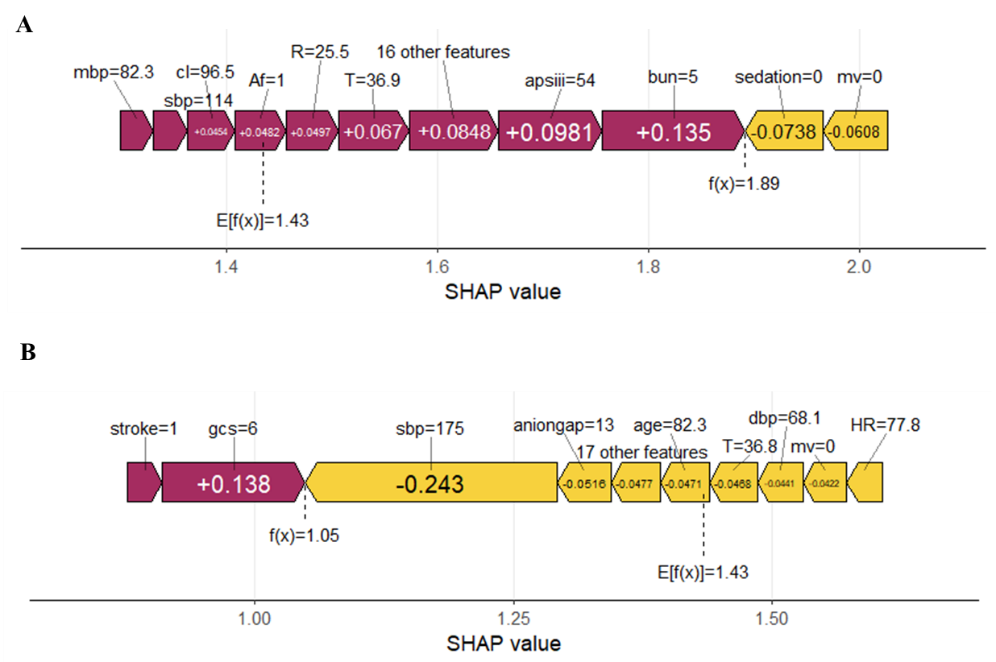


**Fig. S2** The interpretation of the XGBoost model prediction results with two individuals using the SHAP force analysis. The purple arrows indicate a higher risk of delirium, while the yellow arrows represent a lower risk. The f (x) denotes the prediction on the SHAP scale, whereas E[f(x)] refers to the baseline SHAP value. The length of the arrows visually represents the degree of im-pact of the prediction outcome, with longer arrows indicating more significant effects. MBP mean blood pressure, SBP systolic blood pressure, Cl chlorine, AF atrial fibrillation, R respiratory rate, T temperature, APSIII the Acute Physiology Score III, BUN blood urea nitrogen, MV mechanical ventilation, GCS Glasgow Coma Score, HR heart rate.
